# The Impact of the COVID-19 Pandemic on Occupational Stress in Restaurant Work: A Qualitative Study

**DOI:** 10.3390/ijerph181910378

**Published:** 2021-10-02

**Authors:** Julia F. Lippert, Mackenzie B. Furnari, Charlie W. Kriebel

**Affiliations:** 1 College of Science and Health, DePaul University, Chicago, IL 60614, USA; ckriebel@depaul.edu; 2 School of Public Health, University of Michigan, Ann Arbor, MI 48109, USA; mackenziefurnari@gmail.com

**Keywords:** restaurants, occupational stress, COVID-19

## Abstract

The economic downturn due to the COVID-19 pandemic disproportionately impacted the food service industry—one of the largest workforce sectors in the United States. The purpose of this qualitative study was to explore the occupational stressors experienced by restaurant and food service workers during the COVID-19 pandemic through a detailed assessment of their lived experiences. Thematic analysis was used to identify patterns within data from sixteen semi-structured interviews with people employed or recently employed in the restaurant industry during July of 2020. Five themes were highlighted including fear of being exposed to the COVID-19 virus while working under inadequate safety policies, job insecurity, inconsistent pay and hours and a lack of health benefits and paid time off, all of which increased occupational stress and led to uncertainty if respondents would return to the restaurant industry. Hardships associated with the pandemic were mitigated by the support and connections fostered by the communities built within the restaurants. Results led to several recommendations to address the social and economic contributors to occupational stress at the structural and population levels which can be used in the current and post-pandemic workplace.

## 1. Introduction

In March 2020, the COVID-19 pandemic spread to the United States triggering shelter in place orders that largely shutdown the U.S. economy and resulted in a 22% to 60% decrease in the national Gross Domestic Product [1,2]. This economic downturn disproportionately impacted the service industry which prior to the pandemic was one of the largest workforce sectors in the nation with roughly 13.5 million restaurant related jobs. In April 2020, unemployment rates in the leisure and hospitality industries were as high as 39.3% [3,4]. The food service industry has lost nearly 3.1 million jobs and more than 110,000 restaurants have or are projected to permanently close due to the economic fallout caused by the pandemic [5,6].

The pandemic and the associated economic impacts have led to unprecedented levels of occupational stress and adverse mental health outcomes [7]. Prior to the pandemic, 8 out of 10 people reported experiencing stress while recent reports show as high as 94% of workers are experiencing stress [8,9]. Workplace stress is a major factor in 80% of occupational injuries and workers with high stress have on average 46% higher health care expenditures resulting in an estimated loss of USD 200 billion annually in absenteeism, lost productivity, and health care costs [10,11,12].

Various studies have linked occupational stress in the food service industry to excessive workloads, lack of job control, and variable pay and schedules [13,14,15]. With mass layoffs and increased sense of insecurity among employees, several studies have shown that the pandemic is exacerbating these stressors [2,16,17,18]. Seventy-five percent of frontline workers reported that the pandemic has negatively impacted their mental health and as the economy slowly reopens, occupational stress among frontline workers in the restaurant industry continues to grow [19,20]. This is due in part to confusion about what health and safety protocols are recommended and the differential enforcement of these protocols [21]. This, coupled with a lack of attention to workplace stress in general, has led to negative mental health effects in restaurant workers [22].

It is important to identify salient contributing factors to occupational stress to inform health and safety protocols in the reopening efforts. The goal of this study was to explore the occupational stressors experienced by restaurant and food service workers during the COVID-19 pandemic.

## 2. Materials and Methods

This study intended to collect data on the changes to occupational stressors in response to the COVID-19 pandemic. The aim was to identify common themes in the responses of participants through semi-structured interviews. A qualitative approach is appropriate for under-researched areas such as this when data can capture details of the lived experiences of the study participants [23]. This was an attempt to better understand the occupational health impacts of the pandemic and gain insights into the factors associated with stress. This study was therefore exploratory because the events of the COVID-19 pandemic were unpredicted. Interviews were conducted by researchers with experience and training in qualitative data collection and research ethics and the study was approved by the authors’ Institutional Review Board for human subjects’ research.

### 2.1. Data Collection

A total of sixteen interviews took place between June and July of 2020. Participants were selected through purposive sampling; all participants had to be employed at a restaurant at the time of the COVID-19 stay-at-home order. Thirty-nine individuals were sent the invitation and 41% (*n* = 16) responded. All individuals interested in participating were interviewed.

Semi-structured interviews using a research guide (Appendix A) consisting of nine broad questions with between one and three follow-up questions for each topic and several probing questions. The general topics covered were employment status and the financial impact of the pandemic, changes to health status, contributing factors to these impacts, and thoughts on the future of work in the restaurant industry. A brief demographic survey was given including age, race/ethnicity, annual income and wage, gender identity, and work position. Interviews averaged 27 min in length. All interviews were conducted through Zoom and recorded and then transcribed verbatim.

### 2.2. Data Analysis

Thematic analysis was used to identify patterns within worker experiences during the COVID-19 pandemic. Thematic analysis is a process in which data are reviewed several times to develop and revise a set of themes within the data [24]. Qualitative data were broken down into excerpts and given a ‘code’ that summarized the major idea. The lead researcher read all of the transcripts and generated an initial code book and additional researchers coded the excerpts with a subset of codes. Each excerpt was coded by two researchers independently to compare and validate the interpretations. The web application software Dedoose (Los Angeles, CA, USA) was used to track excerpt coding. When disagreements among codes emerged, coders discussed the disagreement until consensus was reached.

## 3. Results

### 3.1. Participant Overview

A total of sixteen (16) people were interviewed (Table 1). All participants were working in Chicago, Illinois prior to the pandemic. At the time of the interviews, stay-at-home orders were in their fourth month. Chicago had temporarily moved into ‘Phase 4’ of the reopening plan with restaurants permitted to have limited indoor dining and unlimited outdoor dining.

Almost half of the participants were laid off (*n* = 7) with the remaining participants employed either at a different job (*n* = 3) or employed at the same job (*n* = 6). The majority of the participants were making less money (*n* = 9) regardless of their employment status and were receiving unemployment insurance benefits (*n*= 10). Three participants did not have health insurance, five participants had employer-sponsored health insurance, three paid for insurance out of pocket, and five were on public insurance.

All but two of the participants (88%) reported having more stress during the pandemic but only half (*n* = 8) said they were having difficulty coping with their stress. All of the participants said that their mental health was worse since the pandemic but when asked to report on their mental health, few of the participants (*n* = 4) said they had “bad” mental health and most reported that their mental health was “OK” or “Good”. Eleven participants reported that their physical health was the same or improved since the pandemic and twelve reported good physical health.

### 3.2. Thematic Analysis

There was a total of 206 excerpts identified as having relevant content across the 16 interviews with an average of 13 excerpts per interview. Five core themes and seventeen subthemes were identified and presented in Table 2 with corresponding excerpts or quotes from the interviews. Excerpts are also identified here within as parenthetical statements.

#### 3.2.1. Community and Social Outlet

The idea that restaurants are a social outlet was mentioned by all but one of the participants. The workplace was described not only as a place for socializing with customers and coworkers, but also a social support system particularly during the pandemic. There was stress associated with the loss of this community due to the shutdown of the restaurant industry.

Most respondents (*n* = 9) discussed their work as a social place and one that they enjoyed because of these connections. For many, this was their attraction to the restaurant industry in general. Respondents explained that work never seemed like work and was fun. This community was shut down along with the restaurant industry and respondents discussed how they felt disconnected and isolated and missed their coworkers and regular customers (3a, 3b). Some respondents felt solace in being part of a community that was suffering through similar challenges while others felt competition and stress from so many people with similar skills being unemployed (2a, 2b). Participants (*n* = 6) that maintained communication with their managers and coworkers even in an informal manner were more comfortable returning to work and had a more positive experience (1a).

Many restaurants held food pantries to supply food or meals to their staff and some raised funds through public donations. However, a number of respondents felt uncomfortable taking the funds explaining that there were people that were worse off than them. One bartender even donated to support out of work people regardless of being out of work herself. Some restaurants also provided financial support through pay cuts for management and owners and by extending health care coverage. This fostered a positive connection and furthered the sense of loyalty within the restaurant. Participants (*n* = 3) that had this support said it impacted their decision to return to work.

#### 3.2.2. Ethical Responsibility of Restaurants

The ethical responsibility of restaurants to their employees was mentioned by 14 of the 16 participants. This responsibility ranged from providing safe workplaces to general support for employee wellbeing.

There was a lack of clarity and communication about policies and procedures to prevent exposure to the COVID-19 virus, what steps were put into place, and how they would be enforced. There was conflicting and confusing information from federal agencies, local politicians, and the restaurants in which they worked (4a). Participants (*n* = 7) said this increased their stress and impacted their desire to return to work. However, when there were clear and effectively communicated standard operating procedures participants felt less stress (5b).

The responsibility of enforcement of the safety protocols was unclear and often fell to the servers. Participants (*n* = 7) found it difficult to enforce rules while at the same time providing a hospitable environment and this added to the stress they experienced at work. Respondents discussed the problematic nature of “policing” the behavior of customers (7a). There was also a lack of consistency with how the restaurants were complying with city-wide mask mandates and capacity recommendations (4b). Some participants (*n* = 8) perceived that this was financial and that the restaurant owners were putting money above their safety (5a).

Participants (*n* = 4) felt job insecurity. This precariousness seemed to be present even before the pandemic as respondents rationalized their feelings as something they’ve always felt. There was a sense that those brought back were the “chosen” few but remained easily replaceable. Participants said that they would be fired if they questioned the standards that the restaurant was implementing, or the hours and wages being offered. This made some participants look for other forms of employment (6a).

#### 3.2.3. Pandemic Related Health Concerns

Twelve participants (75%) had health concerns related to the pandemic creating fear and anxiety. They also reported physical and mental health impacts from elevated stress working as frontline and essential workers.

As an essential worker, the inability to work from home increased occupational stress for some respondents (*n* = 3). Those that had returned to work said they were overwhelmed by how many people they had to interact with and their lack of control over their work environment. In addition, participants discussed the difficulties associated with being a frontline worker without the protections that other industries were afforded (9b, 9c). Participants feared becoming infected with COVID-19. One participant said they felt that it was “only a matter of time.” This stress was compounded for participants that were uninsured (8a).

Participants discussed feelings of burnout indicating that they had been operating under stress for a prolonged time and losing their ability to tolerate the stress. Stress was related to the changing phases of the shutdown with uncertainty about when and if the restaurants would open and at what capacity and the resulting financial instability. Stress (*n* = 5) was also due in part to negative customer interactions that could easily escalate to yelling and even physical altercations (9a).

#### 3.2.4. Wage and Hour, Benefits, and Paid Time Off

Pay, benefits, scheduling, and paid time off was mentioned by all but one of the participants. Participants noted reduced wages, inconsistent pay, and fewer hours due to low-capacity seating and other policies that restaurants were implementing. Participants also highlighted the general lack of benefits such as employer-sponsored health insurance and paid time off which impacted their health and stress.

For those participants (*n* = 9) that had returned to work, their hours and pay were less because of reduced staffing and seating capacity while the workload increased due to the additional cleaning and safety measures. All of the participants were making less money or thought they would when they went back because of less tips (11a, 11b).

Lack of health insurance caused stress for some participants (*n* = 9). One participant calling the lack of insurance “dangerous” during a pandemic and some respondents said it impacted their decision to return to work. Lack of insurance was built into the structure of the industry and one participant explained that restaurants often kept staff part-time to avoid paying for health insurance. In addition, participants were concerned about not having paid sick days if they were to get sick as they would also be out of their pay for that time. Participants pointed out that there was a lot of pressure to work while sick because of this and they often did not seek medical care due to cost (13a, 13b).

Financial insecurity because of low wages caused challenges for participants trying to balance their many demands. One mother said that she had to choose between food, rent, and health insurance and another parent considered filing for bankruptcy in anticipation of continued economic hardships. Many participants (*n* = 7) mentioned government support was keeping them financially solvent and some made more money than had working in a restaurant (10a, 10c). These additional unemployment benefits were set to expire in July of 2021 shortly after the interviews were conducted and the loss of that financial security was stressful for many participants (17a).

Modifying the tipped wage structure was discussed with proponents (*n* = 3) and opponents (*n* = 2). One server said that working for tips “can almost drive a person crazy” explaining that having a consistent wage would help reduce his stress. Another server echoed these concerns saying that she felt that she was always struggling even though she was working every day. Regardless, there remained a sense that some servers would still prefer a tipped wage to a set minimum wage because they could make more money (10b).

#### 3.2.5. Thoughts of the Future

All but one of the participants talked about their considerations for the future. There was uncertainty around returning to work both in if they would return and when and what type of environment to which they would return. Second, financial instability effected whether they would return to the restaurant industry in general.

In July 2020 when these interviews were conducted, the second wave of the pandemic was beginning. Therefore, respondents (*n* = 5) discussed being unsure of their future in the restaurant industry. Some had a place “saved” for them while others were less sure if they would have a position with which to return. Those that were returning were paid less either due to lower tips, less hours, or having to pool their tips with the entire staff including in some cases salaried employees. This uncertainty and lower pay added to respondents’ anxiety about what would come (14a, 15a).

The majority of respondents (*n* = 10) were not returning to their previous job or contemplating leaving the restaurant industry. The reason for this switch varied from not feeling comfortable with safety protocols to perusing a different career. Others felt that there was no other position they could find and worried the restaurant industry would change for the worse. Participants discussed not wanting to “put all their eggs in one basket” and explained that the lowered capacity would result in less hours and fewer tips. There was also a feeling that the industry would change due to the lack of customer interaction (14b, 15b, 16a). Nevertheless, some respondents (*n* = 5) wanted to return to work as they missed customer service and their connections within the restaurants (16b).

When asked directly for suggestions of how the restaurant industry should change when reopening, the participants generally pointed to structural changes. Half of the participants suggested more clear health and safety precautions, better adherence to the protocols, and clarity in the enforcement. Five participants suggested higher wages or less reliance on tips and five participants suggested providing employer-sponsored health insurance.

## 4. Discussion

The lived experiences of restaurant workers outlined here highlight the financial and emotional stressors at the height of the COVID-19 pandemic. Our thematic analysis detailed concerns such as fear of being exposed to the COVID-19 virus while working under inadequate safety policies, job insecurity, inconsistent pay and hours, and a lack of health benefits and paid time off, all of which increased occupational stress. There was also uncertainty if these workers would return to the restaurant industry and what type of environment to which they would return. But ultimately, the most unique aspect was the community built within the restaurant between the staff, owners, and customers which kept people supported and connected during these unprecedented times.

Our findings are consistent with the science of occupational stressors which point to two distinct areas of what causes job stress: worker characteristics such as a workers ability to and style of coping and working conditions such as the workload, working conditions, and organizational structure [25]. Theoretical models of workplace stressors, although varied, attribute elevated stress to an imbalance between inputs (demands, costs, efforts) and outputs (gains, control, rewards) [26,27]. Recent studies of the restaurant and hospital sectors have shown that organizational practices such as increasing workloads without increased compensation and lack of transparency in revised operating procedures during the pandemic had led to more job insecurity, lowered job satisfaction, occupational stress, emotional exhaustion, and less organizational commitment [28,29]. Similarly, our participants reported decreases in job control with the continued shutdowns and lack of clarity in health and safety protections, decreases in rewards with less and variable pay and hours while at the same time experiencing increased work demands due to understaffing, increased duties, and efforts expended towards controlling customer behavior. Increasing social support has been shown to mitigate the effects of these imbalances which was also seen in our findings as the community aspects of restaurant work alleviated stress for many of our participants [30]. Finally, our findings were consistent with the understanding that role conflict and role ambiguity leads to higher levels of stress [31,32]. Our findings showed a disconnect between the expectations of the work prior to and during the pandemic and little if any effort to clearly define the new operating procedures and the responsibilities of enforcement.

These findings contribute to occupational health research by documenting perspectives on which factors within the restaurant are associated with elevated stressors. The factors we found such as the way individuals are paid, requirements for benefits, and financial, emotional, and physical stressors are inherent in the way restaurants have historically operated [33]. These structural components of work contribute to the occupational health disparities we see particularly in low-wage occupations such as food service [34,35,36]. They have been exacerbated during the pandemic adding to the existing disparities in occupational and mental health [22,37,38,39].

The role the pandemic has had on occupational health points to control strategies for the current and post-pandemic workplace. It is most effective to eliminate occupational hazards and focus on the social determinants of health which have broad and sustained impact [40,41]. Therefore, our recommendations for occupational health practice are to address social and economic contributors.

We recommend federal level safety protocols for protecting workers from COVID-19 which are transparently and effectively communicated. Compliance with these protocols should be monitored by restaurant management as opposed to staff. Several federal agencies have released guidance, however as we found and others have shown, implementation and enforcement of these guidelines is not mandated and often falls on restaurant staff [42,43,44]. Removal of the ambiguity in public interfacing procedures and policies will improve not only the occupational health and safety of restaurant workers but will also support the post-pandemic economy specific to the restaurant sector [45].

To address the financial insecurity and associated stress that participants reported, we recommend that the additional unemployment insurance is extended through the pandemic and the subminimum tipped wage eliminated. Our findings and recent studies of stress perception in chefs showed that threats to or the perception of insufficient financial support from the government led to elevated stress [46]. A recent report from the Congressional Budget Office showed that increasing the minimum wage would lift working families out of poverty and lessen the racial and gender wage gap [47,48]. Raising wages can also benefit productivity and lower turnover leading to increased revenue for restaurants [49,50].

We recommended the expansion of employer-sponsored health insurance and paid sick time as it is imperative during this global pandemic. Our respondents reported having little choice but to risk their health and the health of the public by putting off medical care or going to work sick. Employer-sponsored coverage is not required for all restaurants nor is there a federal requirement for paid sick leave. These exceptions should be eliminated or the public options for health insurance extended to include more low-income earners. During the pandemic it was found that as many as 46% of uninsured adults avoided medical care because of cost and between 13% of frontline workers lack health insurance-restaurant workers having one of the highest rates of uninsured individuals [3,51]. These workers need social support to protect themselves as they provide for us as essential workers [52].

Finally, we recommend that the community created within the restaurant industry be leveraged into a network of support. Although restaurants do not fit within the traditional worker union model, there are several worker led organizations that have provided aid through the pandemic by giving financial support and advocating for enhanced worker protections [53,54]. Workers that were connected to an organization such as these were able to negotiate better safety protections, hazard pay, and work-share agreements that allowed for more people to stay employed [55]. These organizations whether formal or informal can be powerful assets to the restaurant community and organizational support has been shown to mitigate occupational stress [28].

There are limitations to this study. The study population represents a small fraction of restaurant workers and is therefore limited in its generalizability. Although small, our sample does represent a cross-section of the restaurant industry in a major urban market. Selection bias may be present exaggerating the observed effects. The sample size is relatively small for an interview based qualitative study however due to the narrow scope of the topics covered and the homogeneity of the work experiences, these data still allowed for data saturation to be achieved. Finally, in an attempt to capture a cross-section of the pandemic’s effects, we collected data within a finite timeframe which didn’t allow for additional data collection if data saturation was not achieved.

Despite these limitations, this study is among the few qualitative studies to report on the experiences of restaurant workers during the COVID-19 pandemic. Qualitative data can describe the social context that quantitative data often lacks and is becoming an increasingly important tool in occupational health research [56,57]. Qualitative methods are important in documenting the lived experiences of workers and importantly in documenting the psycho-social responses of workers which have been underrepresented in research assessing the toll of the COVID-19 pandemic [58].

## 5. Conclusions

This is one of the first studies to report on the stressors associated with restaurant industry during the COVID-19 pandemic. Results show that the restaurants are unique in many ways from the pay and benefits structure to the differential impact of the COVID-19 pandemic and the community that it fosters. These factors have led to challenges for restaurant workers during the restaurant shutdowns and subsequent reopening including financial and employment insecurity, health-related anxieties, and elevated stressors from ambiguous and sometimes dubious practices. These findings have implications for the reopening of our economy and the restructuring of the restaurant industry in response to the changing needs of its employees.

## Figures and Tables

**Table 1:** Participant overview of restaurant workers interviewed (*n* = 16).

Sample Characteristics	*n*	% (Range)
Age (years)		
Mean ± Standard Deviation	35 ± 10	23 to 61
Median	33	
Female	9	56
Race/Ethnicity		
White/Caucasian	6	37.5
Black/African American	4	25
LatinX	2	12.5
Asian	2	12.5
Multiracial	2	12.5
Position		
Server/Bartender	12	75
Cook/Chef/Food Preparation	3	19
Manager	1	6
Annual Income		
USD 10,000–19,000	4	25
USD 20,000–29,000	1	6
USD 30,000–39,000	4	25
USD 40,000–49,000	4	25
USD 50,000–59,000	1	6
≥USD 60,000	2	13
Base Wage without tips (U.S. Dollars)		
Mean ± Standard Deviation	USD 7.68 ± 4.56	USD 4.65 to 22.00
Median	USD 6.40	

**Table 2:** Summary of themes generated from semi-structured interviews.

Themes	Subthemes with Corresponding Excerpts (Participant Race/Ethnicity, Gender, Position)
Community and Social Outlet	The workplace as a social outlet and support system “It just actually created more of a community. And whenever I would get frustrated with my own family, I would be able to talk to my coworkers who were affected in the same way.” (Black, Female, Bartender) “Employees, and I’ve got a really cool crew to work with. Hours, they’re okay, money’s alright. It’s just part time, so. It’s really just the atmosphere, it’s kind of family oriented, people look out for each other, and it’s fun. Work isn’t always work.” (Multiracial, Male, Server) The industry as a whole was affected by the pandemic “We were the first to lose our jobs. We’re gonna be the last to get them back.” (Black, Female, Server) “[I]t was definitely a challenge because everyone was unemployed, and there’s not really anything you can do, necessarily, to get a different job, ‘cause all the restaurants are shut down. But then, at the same time, it potentially made it a little bit easier for unemployment and stuff, because there’s not really the option to work. And even if you are looking for work, it’s just not out there.” (White, Female, Server) Stress related to loss of community “[I]t was really difficult at first, you know, not having that connection and being able to see those people.” (Asian, Female, Server) “I wanna be leaving the house, I wanna be around, you know, the people that I work with, you know, I miss my regulars.” So the social part of it, I think, is like a driving force for me to want to go back to work.” (Multiracial, Female, Server)
Ethical Responsibility of the Restaurants	Steps to protect employees from the COVID-19 virus “I think it’s easier for there to be a consistent protocol. So every restaurant kind of has to follow the same guidelines I think that’s helpful, but I feel like most places and people that work there are doing everything they’re supposed to do.” (White, Female, Bartender) “I think if everybody had a distinct enforced rule and everyone, every single restaurant, every single bar followed this law, there could be a chance at it working, but no one’s enforcing these.” (White, Male, Bartender) Transparency and communication about standard operating procedures “[T]he ones I’ve seen that break the rules, they’re just trying to get their money back up to the way it was, and they don’t really care about wellbeing as much.” (LatinX, Male, Server) “And so our owners were like informing us, sending us news articles and all this stuff. And then just kind of like sharing like a general kind of like what the fuck is happening kind of situation like with people. How to share like kind of like the burden of like trying to comprehend what is the future, what’s going to happen.” (Asian, Male, Server) Job protections and support from management and restaurants owners “[I]t’s so easy to, like, fire people or lay them off in the restaurant industry… [Y]ou can just let go of your staff and then hire a whole new one, if you wanted to. So I don’t know, that just seems a little wrong, but I don’t know how to fix that.” (White, Female, Server/Bartender) Policies related to customer interactions “I think the focus is on protecting customers, and I think that’s just, like, a cultural thing in a lot of service industry, like, a lot of different bars and restaurants.” (Multiracial, Female, Server) “There’s certain rules that we enforce, and that’s part of what adds more to my duties and to the stress. It’s a lot more stress, policing people when they come in.” (Black, Male, Manager)
Pandemic Related Health Concerns	Fear and anxiety of being exposed to the COVID-19 virus through work “And like for a lot of us to it’s like if we get sick, what are we supposed to do? Like we can’t go to work then, we can’t work from home. It’s like, yeah, you literally are just out of a job again. And so I know like that’s like a concern even like for me like I don’t want to get sick and then not be able to work. Because then I’m not making money.” (Asian, Male, Server) Stress while working as essential workers “We’ve had some struggles. I’ve had some people literally want to fight me, because I told them you can’t come in here like that. And they get so frustrated. So everything that happens like that, it comes back and affects you.” (Black, Male, Manager) “Man when everybody else was down we were up, we were the ones that was doing everything, making sure everything got across the board. There was, there was no COVID relief funds for us. There was no health benefits for us. There was no up-raise and pay rate or salary.” (Black, Male, Server) “Essential workers, yeah, we’re essential if like no thank you, no pat on the back, like for real. Let me feel like I’m taken care of. Give me pride, give me assistance to do my job better. I gave my best.” (Black, Female, Server)
Wage and Hour, Benefits, and Paid Time off	Pay scale including tipped wage and policies around pay “It just shows you how shitty the pay is if you can make more in unemployment.” (White, Male, Line Cook) “Because that’s one of the things like with tipped wages like a lot of the time your experience does give you an edge and does end up increasing your wages naturally. But if you were to move away from a tipped structure and you would need to see that reflected still like you would in other jobs.” (White, Female, Server) “Yeah, the financial security that was already kind of iffy being a bartender/server, ‘cause if you had a good night, you made money. If you had a bad night–so you never quite you could gauge what you would kind of make in a week.” (Asian, Male, Server) Lack of consistency in pay and hours in general but also due to the impact of the pandemic “I’m doing more jobs than before, and less hours and less pay, still, you know, we have the place open.” (LatinX, Female, Executive Chef) “Because there’s so many hands in the pot and because people are so overworked, there’s no way that we can go back and try to, on top of all our duties, be janitors or just sanitize everything.” (Black, Female, Server) Financial insecurity due to changes in hours, layoffs, and fear about losing job “So some other friends that are going back to work have told me how they’ve worked the same shift and only made $100.00 where they used to make $500.00 or $600.00.” (White, Male, Bartender) Lack of benefits such as employer-sponsored health insurance, sick days, and paid time off “[E]veryone should have health insurance, I think that should never be like a concern. So I think especially in the restaurant industry too, I think that’s so crucial. Because it’s such a hard industry in general and I feel like sometimes it can become like toxic in like mental, emotional, physical ways. Like no one should be coming into work sick because they’re worried about like not being able to pay their bills, so.” (White, Female, Food Prep) “No one should have to feel the pressure of having to come to work sick or to not–to miss work just because not do things because they have to go to work, essentially thing. So I want to see that change.” (Black, Female, Server)
Thoughts of the Future	Unclear future in the short term with reduced staffing and closures “And also I’d say the uncertainty and the back-and-forth of like, “Okay well we’re going to open now” and then we’re not and now we’re like half-open and now we’re back to not open.” (White, Female, Bartender) “Well now, this is like the new normal. So this is the new baseline. And we don’t know where it’s going to go.” (Black, Male, Manager) Returning to the restaurant industry in general “I’m like I’m just surviving, I’m not thriving. I don’t have insurance, I don’t have like job security. [I]f it’s slow I could be sent home. I could like go to work and maybe make money or not make money. Who wants to do that? I think I’m maybe a little past it all, maybe time to look into the next steps for sure.” (Black, Female, Server) ““[Y]ou realize that you don’t have a job. That line of work can’t function unless people can be social…I mean, after all this, if I could find another job, I would love to just stop bartending ‘cause I don’t see it ever coming back to what it kind of was, at least not for a year or longer.” (White, Male, Bartender) Returning to the restaurant industry during the COVID-19 pandemic “[I] also now may not go back to working in the restaurant industry because I don’t personally feel comfortable with restaurants being open and like outdoor seating happening. Just because you are putting yourself at risk and like servers especially, I, yeah, I don’t know. I kind of find it crazy that people are like back to serving.” (White, Female, Food Preparation) “I am more likely to go back. Before the pandemic, I was thinking about quitting, but after this, and not knowing how another future employer might handle it, not knowing what’s going to happen with the coronavirus itself, I believe in what they’re doing and they’ve shown up so far.” (Black, Female, Bartender/Server) Receiving support from the government and concern about the loss of safety nets and changes to entitlements “Financially, I’m okay right now, because I was able to get unemployment. If I didn’t have unemployment, I would not be doing great right now.” (LatinX, Male, Server) “And I’m super thankful for the extra 600.00 bucks a week, but really scared ‘cause I’m not gonna be able to afford rent or really anything at the end of the month if this expires.” (Black, Female, Server)

## Data Availability

The data presented in this study are available on request from the corresponding author. The data are not publicly available due to the personal nature of the responses.

## Appendix A. Interview Script

The consent has been read to you. Have you had all your questions and concerns answered? If not, please ask me anything else you would like. If all your questions have been answered, do you provide your verbal consent to be in the research? And do we have your permission to record.

**Topic 1: Employment Status and Financial Impact**

To begin, we’d like to know if you are working and what the circumstances of your work are and how these changes have impacted your financial situation.

1. **What is your current employment status?**
  1.1. Are you in the same job/type of work as before the pandemic?
  1.2. How did COVID-19 affect your employment status?
    1.2.1. Are you still employed but with decreased hours, still employed but with increased hours, still employed but have moved online/remote, no change, other, etc?
2. **How is you doing financially?**
  2.1. Are you making more/less/the same amount of money?
  2.2. How difficult is it for you to pay your monthly bills?
  2.3. Do you feel like you have more financial burdens than other people you know?
3. **How have your financial responsibilities changed since the pandemic?**
  3.1. Are you providing homeschooling due to COVID-19?
  3.2. Are you providing homecare (caretaking of elderly, disabilities, etc.) due to COVID-19?

**Topic 2: Health Status**

The next step is to determine your current health status and how it has changed due to the pandemic. Please answer these questions for your current circumstances.

4. **How is your health since the pandemic has happened?**
  4.1. How is your overall physical health?
    4.1.1. Are you getting enough sleep?
    4.1.2. Are you engaging in physical activities and regular exercise?
  4.2. How is your mental health?
    4.2.1. Are you having more anxiety, depression, etc?
  4.3. Have you or any of your friends or family tested positive for COVID-19?
5. **How is your stress level since the pandemic began?**
  5.1. Has stress made it hard to cope with things in your daily life?
    5.1.1. Have you been more upset or irritated than usual?
  5.2. Has the pandemic made you feel less in control?
    5.2.1. Do you feel like you can’t control the important things in your life?
    5.2.2. Are you less confident in your ability to handle your personal life?
    5.2.3. Do you feel like things are pilling up?
6. **Have you been able to access care that you need?**
  6.1. Do you have health insurance?
    6.1.1. If you lost your insurance was it due to losing your job?
    6.1.2. Are you able to pay for supplemental insurance?
  6.2. How has your engagement with medical care changed?
    6.2.1. Have you been engaging in normal preventative care if needed?
    6.2.2. Do you have access to or have you engaged in mental health services if needed?
    6.2.3. Have you used emergency care if necessary?
    6.2.4. Have you had to put any medical needs aside during the pandemic? If so, why?
  6.3. Have you been able to assess COVID-19 related services?
    6.3.1. Were you able to get tested?
    6.3.2. If you or someone you know tested positive, were they able to get the treatment they needed?
    6.3.3. Were you able prevent disease by doing things such as sheltering in place, wear masks, etc.? Why or why not?

**Topic 3: Contributing Factors and Future Directions**

Now we’d like to hear how working in the restaurant industry may be impacting your experience and we’d like to hear from you about how the restaurant industry should reopen and restructure.

7. **Do you think that working in the restaurant industry impacted your experience during the pandemic?**
  7.1. Were there things about the work that made it easier or harder in the past few months?
  7.2. Is there anything the structure of the restaurant industry (benefits, pay scale, management structure) that made it easier or harder during the pandemic?
  7.3. Does the social structure of the restaurant industry (close knit community) that made it easier or harder during the pandemic?
8. **What resources have you accessed during this time?**
  8.1. Have you gotten unemployment benefits and if so, are you getting enough money to support yourself and your family?
  8.2. Have you applied for and/or received any public funding or grant money?
  8.3. Have you utilized other public resources such as food banks, school lunches, etc?
9. **What are somethings that you’d like to see moving forward?**
  9.1. Will you return to the restaurant industry? Why or why not?
    9.1.1. Are you being asked to go back to work?
    9.1.2. If so, how does that make you feel? Are you happy to go back or do you have fears about getting the disease?
    9.1.3. What are they doing to maintain your safety when going back to work?
  9.2. What about the restaurant industry should change as we reopen etc.?

**Thank you!**

Thank you for participating in the survey. This information will be used to understand how restaurant workers are uniquely impacted by the pandemic. It will also be used by the researcher in their research into how the restaurant industry is structured to support its workers. All of the information will be presented as a summary of the survey data and none of what you said will be connected to you directly.
